# CD3ζ-based chimeric antigen receptors mediate T cell activation via *cis*- and *trans*-signalling mechanisms: implications for optimization of receptor structure for adoptive cell therapy

**DOI:** 10.1111/cei.12216

**Published:** 2014-01-03

**Authors:** J S Bridgeman, K Ladell, V E Sheard, K Miners, R E Hawkins, D A Price, D E Gilham

**Affiliations:** * Clinical and Experimental Immunotherapy Group, Department of Medical Oncology, Institute of Cancer Sciences, Manchester Academic Health Centre, The University of ManchesterManchester, UK; † Institute of Infection and Immunity, Henry Wellcome Building, Cardiff University School of MedicineCardiff, UK

**Keywords:** gene therapy, T cells, therapy/immunotherapy

## Abstract

Chimeric antigen receptors (CARs) can mediate redirected lysis of tumour cells in a major histocompatibility complex (MHC)-independent manner, thereby enabling autologous adoptive T cell therapy for a variety of malignant neoplasms. Currently, most CARs incorporate the T cell receptor (TCR) CD3ζ signalling chain; however, the precise mechanisms responsible for CAR-mediated T cell activation are unclear. In this study, we used a series of immunoreceptor tyrosine-based activation motif (ITAM)-mutant and transmembrane-modified receptors to demonstrate that CARs activate T cells both directly via the antigen-ligated signalling chain and indirectly via associated chains within the TCR complex. These observations allowed us to generate new receptors capable of eliciting polyfunctional responses in primary human T cells. This work increases our understanding of CAR function and identifies new avenues for the optimization of CAR-based therapeutic interventions.

## Introduction

Chimeric antigen receptors (CARs) consist of a tumour-associated antigen (TAA)-specific single chain variable fragment (scFv) fused to a T cell-derived signalling chain. Gene-modified T cells that express such receptors can be activated by immobilized antigen and lyse tumour cells expressing the cognate ligand in a major histocompatibility complex (MHC)-unrestricted manner [1–3]. Accordingly, CAR-based approaches are being explored for the treatment of various cancers as alternatives to adoptive cell therapy using patient-derived tumour-infiltrating or peripheral tumour-reactive lymphocytes. Indeed, clinical trials are beginning to show encouraging results [4], putting CARs on a par with the more developed T cell receptor (TCR) gene transfer regimens [5]. Many researchers in the CAR field have adopted CD3ζ [6] as the chosen signalling domain for the activating moiety. However, the mechanisms that underlie CD3ζ-based CAR-mediated T cell activation are not clear and structural distinctions suggest the possibility of signalling pathways that deviate from those employed by the endogenous TCR. A detailed understanding of CAR-mediated signal transduction may therefore reveal new ways to refine this therapeutic approach and optimize the development of second-generation co-stimulatory receptors [7].

Many of the normal TCR-mediated signalling events are known to take place during CAR-driven T cell activation, including tyrosine phosphorylation of CD3ζ [8], recruitment of ZAP70 [9], initiation of the mitogen-activated protein kinase (MAPK) cascade [9] and induction of nuclear factor of activated T cells (NFAT) [10]. It has also been shown that a cytoplasmic domain-deficient CAR containing the FcεRIγ chain can activate T cells via heterodimerization with endogenous CD3ζ [11]. In addition, we have demonstrated that CAR–TCR interactions and dimerization are a prerequisite for optimal CAR-driven T cell activation [12]. These observations suggest that there is cross-talk between the transgene-encoded CAR and endogenous components of the T cell activation machinery.

In an effort to dissect CAR-mediated signalling mechanisms and inform the strategic design of improved receptors, we generated a series of mutant CARs with modified intracellular signalling domains and/or transmembrane domains [12]. These mutant receptors were used to show that CARs can activate T cells directly (in *cis*) and indirectly (in *trans*). The latter mechanism involved both the CAR dimerization partner and other components of the TCR complex, enabling tailored truncations to the signalling domain without significant loss of function. These observations have implications for the optimization of CAR expression and function.

## Materials and methods

### Antibodies

Anti-human CD3ε (UCHT1), anti-human CD3ζ (K25-407·69), anti-human CD69 (FN50), anti-human TCRVβ8 (JR2), anti-mouse CD3ε (145-2C11), mouse IgG1 (MOPC-31C), mouse IgG2b (MPC-11) and hamster immunoglobulin (Ig)G1 (A19-3) phycoerythrin (PE) conjugates were purchased from BD Biosciences (San Jose, CA, USA). Sheep anti-mouse IgG-horseradish peroxidase (HRP) (polyclonal), goat anti-human IgG-PE (polyclonal), mouse anti-FLAG (M2) and mouse anti-FLAG-PE (M2) were purchased from Sigma-Aldrich (St Louis, MO, USA). The following anti-human monoclonal antibodies (mAbs) were used in polychromatic flow cytometry experiments: (i) anti-CD4-PE-cyanin (Cy)5·5 (S3·5) and anti-CD8-QD705 (3B5) (Life Technologies, Carlsbad, CA, USA); (ii) anti-interferon (IFN)-γ-V450 (B27) (BD Horizon, BD Biosciences); and (iii) anti-CD107a-fluorocein isothiocyanate (FITC) (H4A3), anti-interleukin (IL)-2-allophycocyanin (APC) (MQ1-17H12) and anti-tumour necrosis factor (TNF)-α-PECy7 (Mab11) (BD Pharmingen, Franklin Lakes, NJ, USA).

### Generation of chimeric antigen receptor constructs

All CAR constructs used in this study contain the MFE23 anti-carcinoembryonic antigen (CEA) single chain antibody [13]. The MFEζ CAR has been described previously [14]. MFE.htmζ consists of MFE23 fused to MHC class I truncated extracellular and transmembrane domains (amino acids 274–313) and the human CD3ζ cytoplasmic domain (amino acids 32–142). MFEζ.C2G G-10C is a mutant form of MFEζ with a displaced cysteine residue that permits preferential homodimerization [12]. Introduction of immunoreceptor tyrosine-based activation motif (ITAM) mutations into the CAR constructs was carried out using a site-directed mutagenesis kit (Invitrogen, Carlsbad, CA, USA), according to the manufacturer's instructions (primer details available on request).

### Cell lines

The following cell lines were used: (i) 293T (American Type Culture Collection); (ii) Jurkat E6·1 (European Collection of Cell Cultures); (iii) TCR-β-deficient Jurkat derivative JRT3-T3·5, a kind gift from Dr Reno Debets (University Medical Center, Rotterdam, the Netherlands); and (iv) mouse MA5·8, a kind gift from Dr David Wiest (Fox Chase Cancer Centre, Philadelphia, PA, USA). Jurkat and MA5·8 cells were maintained in T cell medium (TCM), comprising RPMI supplemented with 10% heat-inactivated fetal calf serum (FCS), 0·01 M HEPES, 1% L-glutamine and 0·1% 2-mercaptoethanol; 293T cells were maintained in Dulbecco's modified Eagle's medium (DMEM) supplemented with 10% FCS.

### Retrovirus generation and lymphocyte transduction

The rKat retroviral vector system was used as described previously [15]. Retroviral supernatant was produced from transiently transfected 293T cells using a modified calcium chloride transfection protocol [13]. Medium from 293T cells was passed through a 0·45 μm filter and used to infect cells directly. Typically, 5 × 10^5^ T cells were mixed with retroviral supernatant and 6 μg/ml polybrene, then centrifuged for 3 h at 1200 ***g***. Transductions were repeated on two consecutive days.

### Flow cytometric analysis and sorting

Transduction levels were assessed using CEA.hFc protein generated from transiently transfected 293T cells, as described previously [14]. Basic flow cytometric analysis was conducted using either a FACScan or a FACSCalibur flow cytometer (BD Biosciences) and WinMDI version 2·8 software. Polychromatic flow cytometric analysis was conducted using a custom-modified FACSAriaII flow cytometer (BD Biosciences) and FlowJo software version 9·5.3 (TreeStar Inc., Ashland, OR, USA). Cells were sorted using either a FACSVantage or a FACSAria flow cytometer (BD Biosciences) to obtain populations with equivalent CAR surface expression (generally the top 5%). All cell populations were >90% CAR^+^ after enrichment.

### Polyacrylamide gel electrophoresis and Western blotting

Cells were lysed in 1% CHAPS (3-[(3-cholamidopropyl)dimethylammonio]-1-propanesulphonate) buffer [phosphate-buffered saline (PBS), 1% CHAPS, 0·5% sodium deoxycholate, 2% sodium dodecyl sulphate] containing complete protease inhibitor cocktail tablets (Roche, Indianapolis, IN, USA) for 30 min, then centrifuged through polymer wool to clear the supernatant. Protein concentrations were established using a protein estimation kit (Bio-Rad, Hercules, CA, USA). Reduced or non-reduced samples containing 20 μg of total protein were separated on 10% sodium dodecyl sulphate-polyacrylamide gel electrophoresis (SDS-PAGE) gels, transferred to Hybond nitrocellulose membranes (Amersham Biosciences, Pittsburgh, PA, USA) and probed with mouse anti-human CD3ζ (BD Biosciences) followed by HRP-conjugated sheep anti-mouse IgG (Sigma-Aldrich), both diluted 1:1000 in PBS-Tween/5% skimmed milk powder. Bands were resolved using an ECL Western Blotting Detection kit (Amersham Biosciences), according to the manufacturer's instructions, and the membranes were analysed by exposure to X-ray film.

### CD69 expression assay

Ninety-six-well flat-bottomed non-tissue culture plates were coated overnight with the indicated concentrations of CEA protein diluted in borate-buffered saline (0·015 M sodium borate, 0·15 M sodium chloride, pH 8·5). The plates were then blocked for 1–2 h at 37°C with 2% bovine serum albumin (BSA)/PBS before addition of 1 × 10^5^ CAR-transduced T cells in 100 μl of TCM per well. After incubation for 24 h at 37°C, cells were stained with anti-CD69-PE and analysed by flow cytometry.

### Polyfunctionality assay

Plates were coated with CEA and blocked as above before the addition of 3 × 10^5^ CAR-transduced T cells per well in the presence of brefeldin A (10 μg/ml; Sigma-Aldrich), GolgiStop (0·7 μl/ml; BD Biosciences) and anti-CD107a-FITC. After incubation for 6 h at 37°C, cells were washed in FACS buffer (PBS/1% FCS) and incubated for 10 min at room temperature with LIVE/DEAD® Fixable Aqua stain (Life Technologies). Cells were then surface-stained with anti-CD4-PECy5·5 and anti-CD8-QD705, washed twice in FACS buffer, permeabilized using a Cytofix/Cytoperm kit (BD Biosciences) and stained intracellularly with anti-FLAG-PE, anti-IFN-γ-V450, anti-IL-2-APC and anti-TNF-α-PECy7. Data were collected on a custom-modified FACSAriaII flow cytometer and analysed with FlowJo software version 9·5.3 (TreeStar Inc.). Graphics were generated using the spice software suite [16].

### Statistical analysis

Up-regulation of CD69 expression was analysed using a Kruskall–Wallis test. Half maximal effective concentration (EC_50_) values were compared using one-way analysis of variance (anova). Significance levels are indicated as *P < 0·05, **P < 0·01 and ***P < 0·001.

## Results

### Chimeric antigen receptors can activate T cells via their heterodimerizing native CD3ζ partner

It has been demonstrated previously that CARs containing the transmembrane region of FcεRIγ can mediate T cell activation by heterodimerizing with CD3ζ [11]. This observation suggests that CAR-driven signalling pathways are more complex than first thought. To determine whether the same is true for CD3ζ-based CARs, we generated a panel of receptors with defined interchain interactions. MFEζ consists of the CEA-specific scFv MFE23 fused directly to the CD3ζ signalling chain from the TCR complex, allowing both homodimerization and heterodimerization with endogenous CD3ζ [12]. MFEζ.C2G G-10C differs from MFEζ in that the interchain disulphide bond has been moved from the transmembrane C2 to the extracellular G-10 position. The displaced disulphide bridge eliminates heterodimerization with endogenous CD3ζ, but the capacity to homodimerize and interact with the endogenous TCR is maintained [12]. MFE.htmζ incorporates the human leucocyte antigen (HLA)-A*02 transmembrane domain fused to the CD3ζ cytoplasmic domain; this receptor can only form monomers, and is unable to interact with the endogenous TCR complex [12]. We also generated identical receptors with Y–F (Tyr–Phe) mutations at each ITAM within the cytoplasmic domain, thus making them signalling-deficient [17]. All receptors were cloned with a C-terminal FLAG-tag to permit immunoblot and flow cytometric detection.

**Fig. 1:**
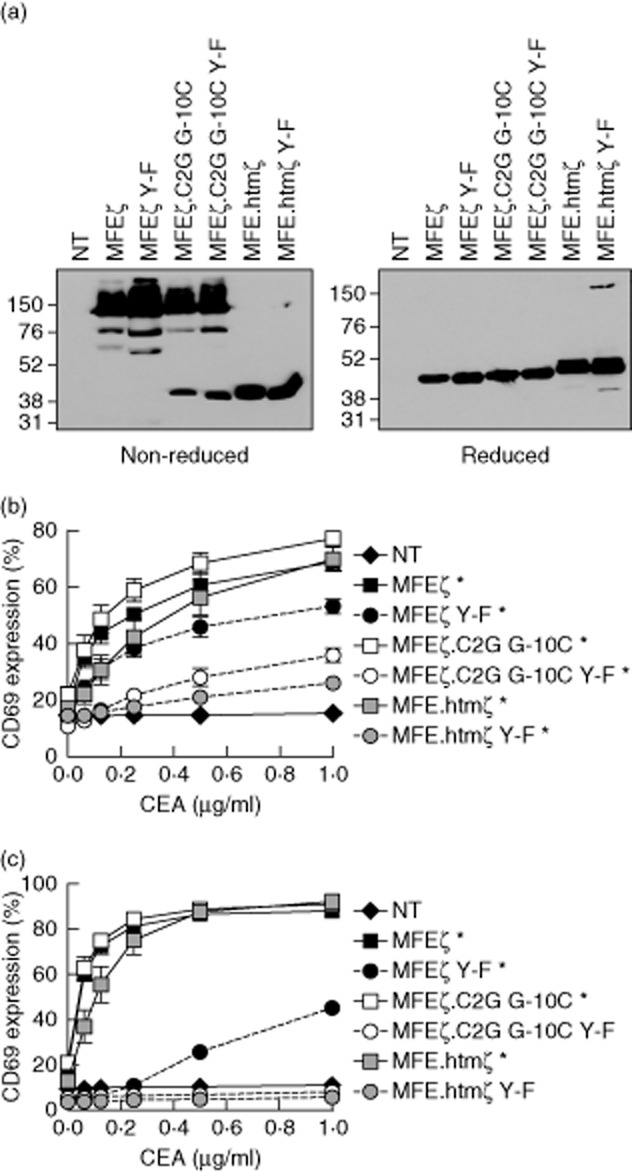
Immunoreceptor tyrosine-based activation motif (ITAM)-mutant chimeric antigen receptors retain a spectrum of functional activity in Jurkat E6·1 and JRT3-T3·5 cells –Jurkat E6·1 cells were transduced with retroviral constructs encoding the indicated chimeric antigen receptors (CARs) and sorted by flow cytometry to obtain enriched populations of CAR^+^ cells. CAR^+^ cells were lysed and subjected to sodium dodecyl sulphate-polyacrylamide gel electophoresis (SDS-PAGE). Transferred proteins were detected using an anti-FLAG monoclonal antibody (mAb). Reducing and non-reducing conditions are shown with molecular weight (KDa) markers (a). Transduced Jurkat E6·1 (b) or JRT3-T3·5 (c) cells were assessed for surface expression of the T cell activation marker CD69 after stimulation for 24 h with the indicated concentrations of immobilized carcinoembryonic antigen (CEA) protein. Significant increases in CD69 expression between 0 μg/ml and 1·0 μg/ml are shown as *P < 0·05, **P < 0·01 and ***P < 0·001 (Kruskal–Wallis test). For activation assays (b,c), arithmetic means of three independent experiments ± standard deviation are shown. NT: non-transduced.

Jurkat T cells were transduced with retroviral vectors encoding the indicated CARs. Immunoblot analysis confirmed the presence of CAR heterodimers and homodimers in MFEζ-expressing cells, homodimers and monomers in MFEζ.C2G G-10C-expressing cells and monomers only in MFE.htmζ-expressing cells (Fig. 1a). Cellular activation in response to CEA was assessed using flow cytometry to measure CD69 up-regulation. This analytical approach in Jurkat T cells closely mirrors CAR-mediated activation via ‘signal 1’ in primary human T cells [12]. MFEζ and MFEζ.C2G G-10C up-regulated CD69 most potently, followed by MFE.htmζ. All three CARs induced significant increases in CD69 expression at CEA concentrations of 1·0 μg/ml (Fig. 1b). The ITAM-mutant CARs responded in the same relative order (MFEζ > MFEζ.C2G G-10C > MFE.htmζ). This result confirms that MFEζ can mediate T cell activation in a CAR-independent manner, presumably via heterodimerization and potentially via interactions with the TCR complex. The observation that MFEζ.C2G G-10C Y–F up-regulated CD69 further suggests the existence of a mechanism beyond heterodimerization with endogenous CD3ζ.

To dissect this phenomenon further, the same experiment was repeated in TCR-β-deficient Jurkat T cells (JRT3–T3·5). These cells have a mutation in the TCR-β locus and, consequently, do not express a complete surface TCR complex [18]. They do, however, express CD3ζ. As in parental Jurkat T cells, CAR-mediated up-regulation of CD69 followed the relative order MFEζ > MFEζ.C2G G-10C > MFE.htmζ, albeit with responses that appeared to be more sensitive compared to the parental E6·1 cells. This latter observation could reflect higher surface expression of CARs transduced into the JRT3–T3·5 line or intrinsic differences in cellular sensitivity to stimulation. The only ITAM-mutant CAR to show any significant activity was MFEζ Y–F (Fig. 1c). This finding suggests that the primary *trans*-activatory mechanism underlying CAR-driven T cell activation occurs via endogenous CD3ζ. However, other components of the TCR are also implicated by the observation that MFEζ.C2G G-10C Y–F lost all activity in this system.

### Chimeric antigen receptor-mediated *trans*-activation is favoured by interactions with the TCR

**Fig. 2:**
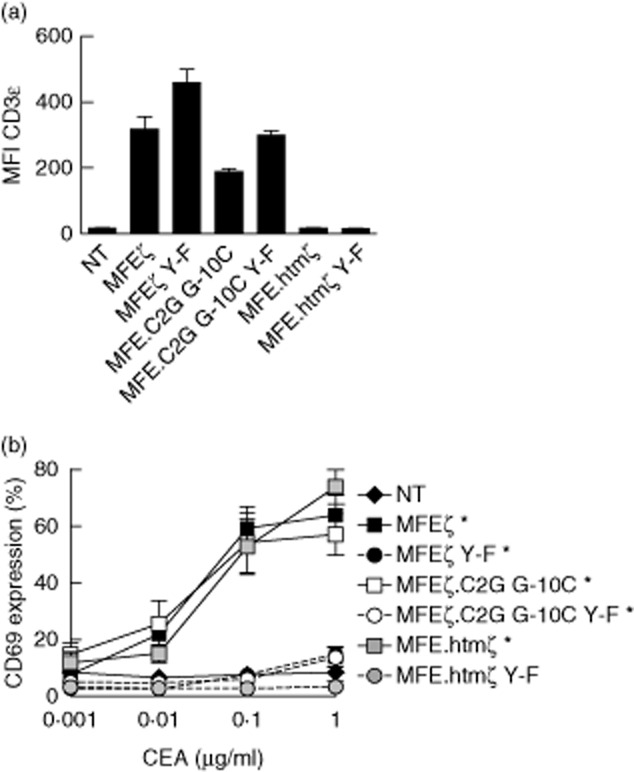
Residual chimeric antigen receptor *cis*- and *trans*-signalling mechanisms permit activity in CD3ζ-deficient MA5·8 cells – CD3ζ-deficient MA5·8 cells were transduced with the indicated chimeric antigen receptors (CARs) and enriched for expression. CD3ε surface expression was measured to evaluate CAR–T cell receptor (TCR) interactions (a). Transduced MA5·8 cells were assessed for surface expression of CD69 after stimulation for 24 h with the indicated concentrations of immobilized carcinoembryonic antigen (CEA) protein (b). Significant increases in CD69 expression between 0 μg/ml and 1·0 μg/ml are shown as *P < 0·05, **P < 0·01 and ***P < 0·001 (Kruskal–Wallis test). Arithmetic means of three independent experiments ± standard deviation are shown. NT: non-transduced.

To elucidate the role of TCR *trans*-signalling events, we conducted the same experiment in the MA5·8 cell line, which expresses all components of the TCR complex except CD3ζ [19]. These cells express low levels of the endogenous TCR, which is up-regulated in response to CD3ζ-based CAR expression; the magnitude of this effect is related to the strength of the CAR–TCR interaction [12,20]. MFEζ (± Y–F) up-regulated TCR expression to the greatest extent, as established by measurement of surface CD3ε, followed by MFEζ.C2G G-10C (± Y–F). In contrast, MFE.htmζ (± Y–F) was unable to rescue surface TCR expression (Fig. 2a). As in the Jurkat system, all receptors with intact ITAMs up-regulated CD69 robustly in response to immobilized CEA. Interestingly, we found that MFEζ Y–F and MFEζ.C2G G-10C Y–F induced significant (*P* < 0·05) increases in CD69 expression, despite the absence of functional CAR ITAMs and endogenous CD3ζ (Fig. 2b). MFE.htmζ failed to up-regulate CD69 expression. These results suggest that CARs can mediate T cell activation via interactions with the endogenous TCR.

### *Trans*-activation permits tailored truncations to chimeric antigen receptor signalling domains

**Fig. 3:**
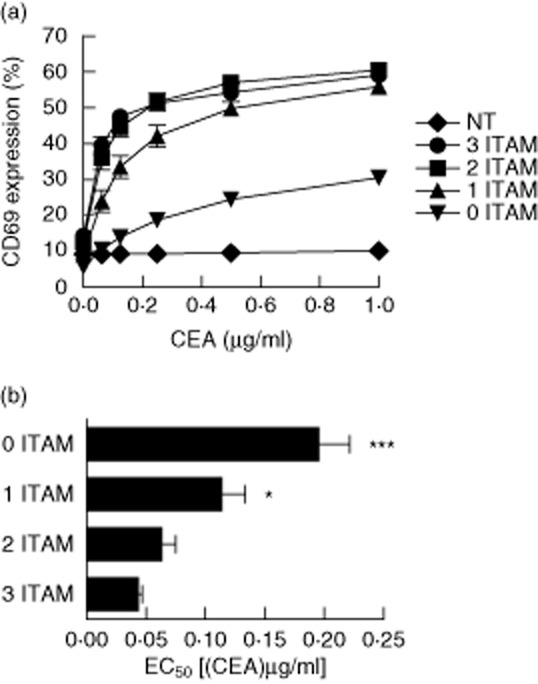
Immunoreceptor tyrosine-based activation motif (ITAM)-truncated chimeric antigen receptors retain functional capacity in Jurkat E6·1 cells – Jurkat E6·1 cells were transduced with the indicated full-length and truncated MFEζ variants and enriched for expression. Transduced cells were assessed for surface expression of CD69 after stimulation for 24 h with the indicated concentrations of immobilized carcinoembryonic antigen (CEA) protein (a) and EC_50_ values were compared by one-way (anova) (b). *P < 0·05, **P < 0·01 and ***P < 0·001. Arithmetic means of three independent experiments ± standard deviation are shown. NT: non-transduced.

The data presented above indicate that CARs activate T cells via multiple mechanisms, the relative potency of which appears to follow the order *cis*-signalling > CD3ζ *trans*-signalling > TCR *trans*-signalling. This led us to hypothesize that we could reduce the size of the cytoplasmic domain without a concomitant loss of signalling capacity. Previous work has shown that the order of ITAM phosphorylation is crucial for T cell activation, such that the membrane-proximal first ITAM is required for phosphorylation of the adjacent signalling motifs [21]. Accordingly, we progressively truncated MFEζ from the C-terminal third ITAM. The antigen responsiveness of these ITAM-truncated CARs was assessed in transduced Jurkat T cells as above. Interestingly, we found that potency was largely unaffected by the lack of the third ITAM. Truncation of the second ITAM, however, reduced antigen responsiveness (Fig. 3a). Receptors lacking all three ITAMs were profoundly impaired in functional terms compared to the wild-type receptor, an observation akin to that seen with the ITAM-mutant receptors tested in Fig. 1. These differences were reflected in measurements of antigen sensitivity, with receptors containing no ITAMs or just a single ITAM having significantly higher EC_50_ values compared to the wild-type receptor (*P* < 0·001 and *P* < 0·05, respectively) (Fig. 3b).

**Fig. 4:**
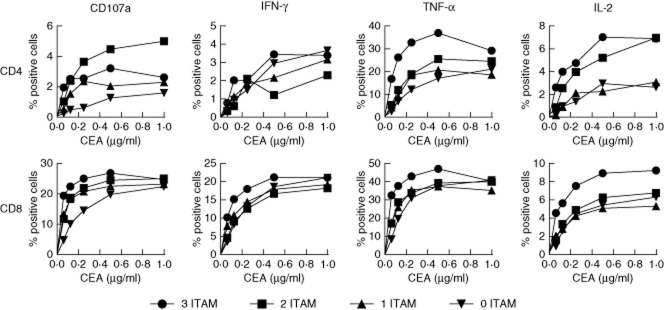
Immunoreceptor tyrosine-based activation motif (ITAM)-truncated chimeric antigen receptors retain functional capacity in primary human T cells – T cells from three healthy donors were transduced with the indicated chimeric antigen receptors (CARs) and expanded *in vitro*. Transduced CD4^+^ and CD8^+^ cells, identified by FLAG expression, were assessed for CD107a mobilization and intracellular production of interferon (IFN)-γ, tumour necrosis factor (TNF)-α and interleukin (IL)-2 after stimulation for 6 h with the indicated concentrations of immobilized carcinoembryonic antigen (CEA) protein. Representative data from one donor are shown.

To confirm these observations, we transduced primary human T cells from three healthy donors with receptors containing no, one, two or three ITAMs. After *in-vitro* expansion, we activated these transduced cells with immobilized CEA and measured intracellular cytokine production and CD107a mobilization by flow cytometry (Fig. 4 and Supporting information, Fig. S1). Regardless of the number of ITAMs, all of the CARs tested elicited all four measured functions in response to CEA stimulation. As expected, response magnitude and antigen sensitivity for the majority of functional readouts followed the hierarchy observed in Jurkat cells, with CAR efficacy dictated by the number of ITAMs. Notably, some functions were impacted more by the loss of ITAMs than others; in addition, this effect varied with T cell lineage. The production of IL-2 appeared to be most sensitive to the loss of ITAMs, especially in CD4^+^ cells. Furthermore, in two of the three donors tested, the three-ITAM receptor was not optimal for CD107a mobilization in CD8^+^ cells. Greater frequencies of CD8^+^ cells responded to CAR-mediated antigen recognition compared to CD4^+^ cells, particularly with regard to IFN-γ production.

**Fig. 5:**
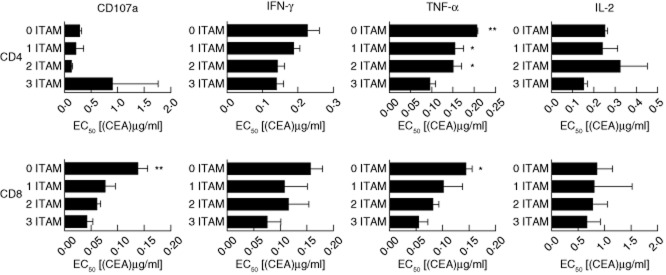
Immunoreceptor tyrosine-based activation motif (ITAM)-truncated chimeric antigen receptors do not show significant variations in antigen sensitivity for most effector functions in CD4^+^ and CD8^+^ T cell lineages – T cells from three healthy donors were transduced with the indicated chimeric antigen receptors (CARs) and expanded *in vitro*. Transduced CD4^+^ and CD8^+^ cells, identified by FLAG expression, were assessed for CD107a mobilization and intracellular production of interferon (IFN)-γ, tumour necrosis factor (TNF)-α and interleukin (IL)-2 after stimulation for 6 h with the indicated concentrations of immobilized carcinoembryonic antigen (CEA) protein. EC_50_ values were calculated using GraphPad Prism software and compared by one-way analysis of variance (anova). *P < 0·05, **P < 0·01 and ***P < 0·001 compared to three ITAM.

**Fig. 6:**
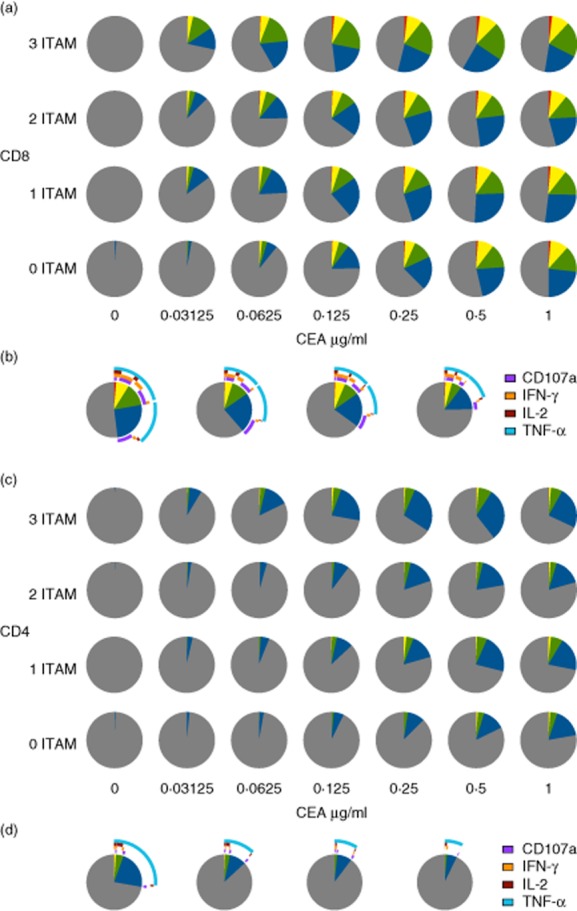
Immunoreceptor tyrosine-based activation motif (ITAM)-truncated chimeric antigen receptors elicit polyfunctional responses in CD4^+^ and CD8^+^ T cell lineages – T cells from three healthy donors were transduced with the indicated chimeric antigen receptors (CARs) and expanded *in vitro*. Transduced CD4^+^ and CD8^+^ cells, identified by FLAG expression, were assessed for CD107a mobilization and intracellular production of interferon (IFN)-γ, tumour necrosis factor (TNF)-α and interleukin (IL)-2 after stimulation for 6 h with the indicated concentrations of immobilized carcinoembryonic antigen (CEA) protein. The functional profiles of CD8^+^ (a,b) and CD4^+^ (c,d) cells were analysed using spice software. Pie charts depict the number of functions as follows: grey, 0; blue, 1; green, 2; yellow, 3; red, 4. Arc graphs indicate the proportion of cells with each combination of effector functions for each CAR (from left to right: three ITAM, two ITAM, one ITAM and no ITAM) at a CEA concentration of 0·125 μg/ml for CD8^+^ (b) and CD4^+^ (d) cells.

To ascertain antigen sensitivity, we calculated EC_50_ values for each function (Fig. 5). Although the maximum response elicited by each receptor varied, we found few differences in terms of antigen sensitivity. Compared to the three-ITAM receptor, the only significant differences in EC_50_ values were observed for CD107a in CD8^+^ cells (three-ITAM *versus* no ITAM, *P* < 0·01), TNF-α in CD4^+^ cells (three-ITAM *versus* no ITAM, *P* < 0·01; three-ITAM *versus* one/two-ITAM, *P* < 0·05) and TNF-α in CD8^+^ cells (three ITAM *versus* no ITAM *P* < 0·05). In addition, we analysed the functional profiles of CAR-transduced T cells using spice software [16]. All CARs tested displayed dose-dependent polyfunctionality profiles within both the CD4^+^ and CD8^+^ lineages (Fig. 6). Notably, polyfunctionality was more obvious in CD8^+^ cells compared to CD4^+^ cells (Fig. 6a,b). Furthermore, there appeared to be a gradual decrease in polyfunctionality at intermediate CEA concentrations as the CARs were progressively truncated, particularly when the final ITAM was removed (Fig. 6b,d). The primary function elicited in CD8^+^ cells was TNF-α production. A large proportion of CD8^+^ cells were positive for TNF-α plus CD107a and/or IFN-γ. However, only a fraction of cells elicited all four effector functions (Fig. 6b). The majority of CD4^+^ cells elicited only a single function (primarily TNF-α); very few elicited two, three or four effector functions (Fig. 6d).

Together with the observations made in Jurkat cells, these data suggest that the third ITAM is largely dispensable for CAR function. Further truncations, however, can have a negative impact on certain effector functions.

## Discussion

There is growing enthusiasm for the use of adoptively transferred immune cells in the treatment of cancer. In part, this is driven by promising work conducted with gene-modified T cells engrafted with tumour-reactive TCRs [5]. Synthetic receptors, such as CARs, circumvent the requirement of TCRs for MHC restriction, a feature that expands their potential use in tailored cancer therapies. It is important, therefore, to establish the mechanisms that underlie CAR-mediated T cell activation. Previously, we showed that CAR–TCR interactions enhance CAR-driven activation of transduced T cells. However, the mechanistic basis for this effect was not elucidated. In particular, it was not clear whether the interacting TCR complex was functioning as a distinct signalling unit or simply facilitating T cell activation in an indirect manner. The present study demonstrates that the TCR complex does indeed act as an independent signalling unit for CAR-driven T cell activation, co-ordinating with signals mediated by the CAR itself and the native CD3ζ signalling chain. Accordingly, the molecular events downstream of CAR ligation are more complex than initially anticipated. Indeed, the ability of CD3ζ-based CARs to interact with components of the TCR complex permits targeted truncations of the signalling chain without undue functional consequences.

Our observations lead to an important question. How do CAR molecules actually transmit signals across the membrane? This issue remains unresolved even for native TCRs. Currently popular torsion-based models have given rise to the idea that the TCR is a mechanosensor [22]. Such models permit conformational changes in the ionically interacting CD3 chains that favour their phosphorylation. In contrast, our data suggest that the opposite is true for CAR-driven T cell activation; i.e. conformational changes in CD3ζ induce structural deformation of other components of the TCR complex. Alternatively, antigen-mediated clustering of CARs may allow phosphorylation of the TCR complex by src kinases. The conformational change model of CD3ζ signalling is supported by our recent observation that CAR function is seriously impaired when the highly vibrational transmembrane L9 residue is mutated [12,23,24]. This hypothesis, whereby activation is driven by conjugation of one of the CD3 chains, is not unrealistic. Indeed, it is in essence the mechanism that underlies the activation of T cells by mitogenic antibodies such as OKT3 and UCHT1 [25].

In agreement with a previous study [17], we found that deleting the third ITAM had little effect on the overall function of CD3ζ chimeras. Conversely, both Chae *et al*. and Geiger *et al*. [17,26] demonstrated that a greater functional deficit accompanied the loss of the two C-terminal ITAMs. However, neither of these studies would have taken heterodimerization into account due to the use of CD8 and MHC class I transmembrane domains. The maintenance of CAR function in the absence of the third ITAM may explain why the FCεRIγ signalling subunit, which contains just two ITAMs, has proved to be a useful alternative to CD3ζ in a number of studies [27,28]. Removing the C-terminal ITAM from the introduced CAR may also reduce CD3ζ-mediated apoptosis, as demonstrated by Lenardo and colleagues [29]. The effects of ITAM removal are likely to be less obvious in our system due to the aforementioned capacity to heterodimerize, which compensates to some extent for the lost signalling capacity. However, the key point is that CAR size can be reduced without loss of function. Together with our recent work identifying a role for TCR–CAR interactions in optimal CAR function, these results highlight the importance of using the CD3ζ transmembrane domain as a component of CARs. In addition, we show here that the capacity of CD3ζ transmembrane-bearing CARs to interact with the TCR complex enables signalling in a CD3ζ-independent manner. Further work is required to determine which components of the TCR are important for this function.

Although IL-2 production appears to be the function most affected by ITAM loss, this does not impinge upon the primary objective of first generation CARs. Indeed, CD28 has a largely synergistic effect on IL-2 production mediated by the TCR. Thus, ITAM-mutant second-generation CARs containing the CD28 signalling module may not show any functional compromise in terms of IL-2 production. A caveat to this may be the use of second generation receptors incorporating the CD3ζ transmembrane region which, although tested and published, have not been compared critically to existing receptors [30].

Herein, we report a comprehensive analysis of T cell effector function elicited by CAR-mediated antigen recognition. Polyfunctional response profiles were observed with both intact and ITAM-truncated CARs, more noticeably in CD8^+^ cells compared to CD4^+^ cells. This latter phenomenon might reflect differential lineage-specific activation thresholds. Alternatively, it could hinge upon the amount of free Lck available for CAR-mediated activation. Indeed, Lck has been shown to associate preferentially with CD4 compared to CD8 [31], potentially restricting its availability in CD4^+^ T cells. Strategies that enhance the polyfunctional effector profiles of CD4^+^ T cells may therefore improve the efficacy of CAR therapy, particularly as it has been established that CAR-engrafted CD4^+^ T cells play a crucial role in tumour eradication [2].

In summary, we show here that CARs incorporating the CD3ζ transmembrane domain can signal via endogenous CD3ζ as well as via other undefined components of the TCR complex. This observation led us to demonstrate that CARs with truncated cytoplasmic domains can still function in both Jurkat T cells and primary human T cells, eliciting polyfunctional profiles in both the CD4^+^ and CD8^+^ lineages. Rather than activation being dependent upon a stoichiometrically equal ratio of CAR to antigen, it seems more likely that the various signalling chains involved upon CAR binding permit one antigen to mediate a network of interchain phosphorylation events. Such a process would explain the high antigen sensitivity of CARs. Moreover, the cross-talk is efficient enough for ITAMs to be deleted from the signalling chain without concomitant functional impairments. This is an important observation because it means that the size of the transgene, or at least the size of the CD3ζ component of next generation CARs, can be reduced without incurring therapeutically relevant loss of function. Such observations open avenues for tailoring the cytoplasmic domain of CARs, removing unnecessary components and in turn replacing them with motifs that enhance both expression and function.

## Supporting information

**Fig. S1.** Immunoreceptor tyrosine-based activation motif (ITAM)-truncated chimeric antigen receptors retain functional capacity in primary human T cells – T cells from three healthy donors were transduced with the indicated chimeric antigen receptors (CARs) and expanded *in vitro*. Transduced CD4^+^ and CD8^+^ cells, identified by FLAG expression, were assessed for CD107a mobilization and intracellular production of interferon (IFN)-γ, tumour necrosis factor (TNF)-α and interleukin (IL)-2 after stimulation for 6 h with the indicated concentrations of immobilized carcinoembryonic antigen (CEA) protein.
