# Supplementary figures and images for: CD3ζ-based chimeric antigen receptors mediate T cell activation via cis- and trans-signalling mechanisms: implications for optimization of receptor structure for adoptive cell therapy
Source: Clin Exp Immunol. 2014 Jan 3;175(2):258–67. doi: 10.1111/cei.12216 (PMC3892417; doi:10.1111/cei.12216)

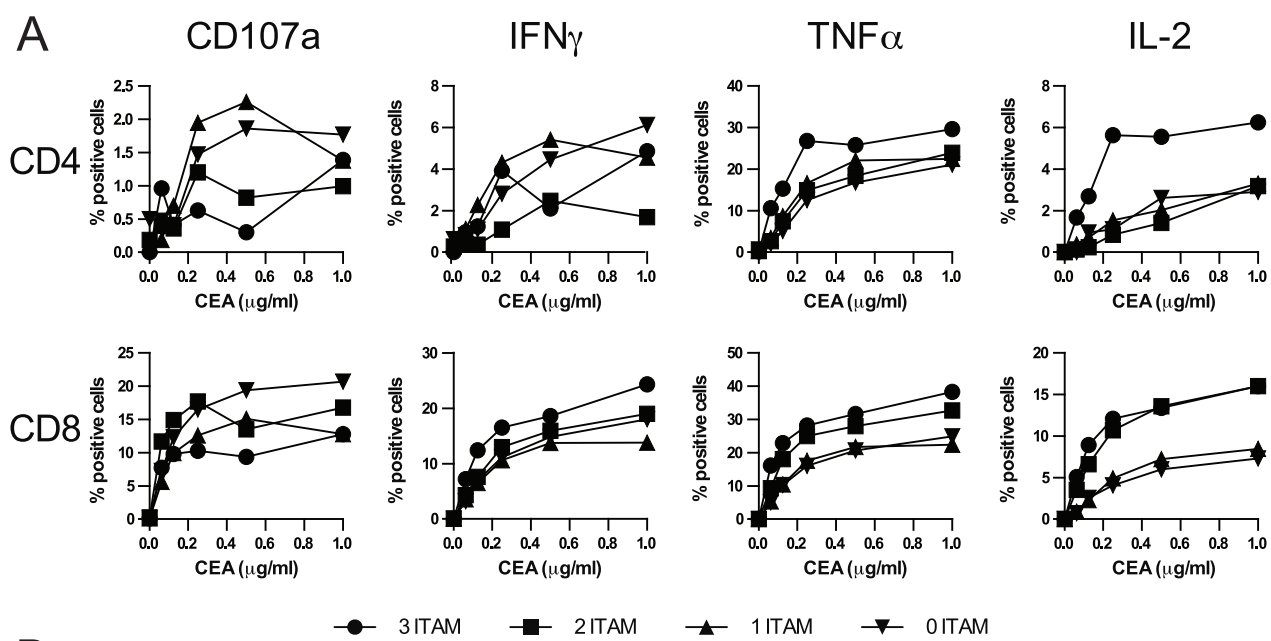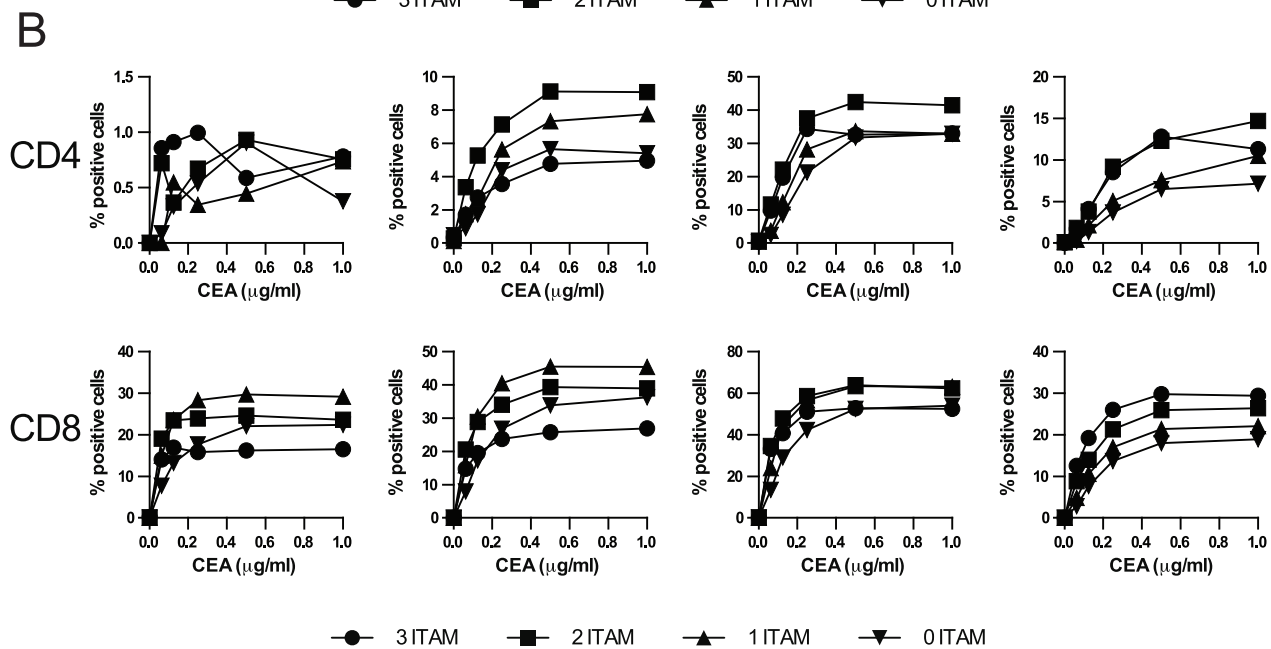

Supplementary figure 1

Supplement: Supplementary file 1 [file cei0175-0258-SD1.pdf]
